# Heparanase Facilitates Cell Adhesion and Spreading by Clustering of Cell Surface Heparan Sulfate Proteoglycans

**DOI:** 10.1371/journal.pone.0002319

**Published:** 2008-06-11

**Authors:** Flonia Levy-Adam, Sari Feld, Edith Suss-Toby, Israel Vlodavsky, Neta Ilan

**Affiliations:** Cancer and Vascular Biology Research Center, Ruth and Bruce Rappaport Faculty of Medicine, Technion, Haifa, Israel; Illinois Institute of Technology, United States of America

## Abstract

Heparanase is a heparan sulfate (HS) degrading endoglycosidase participating in extracellular matrix degradation and remodeling. Apart of its well characterized enzymatic activity, heparanase was noted to exert also enzymatic-independent functions. Non-enzymatic activities of heparanase include enhanced adhesion of tumor-derived cells and primary T-cells. Attempting to identify functional domains of heparanase that would serve as targets for drug development, we have identified heparin binding domains of heparanase. A corresponding peptide (residues Lys^158^-Asp^171^, termed KKDC) was demonstrated to physically associate with heparin and HS, and to inhibit heparanase enzymatic activity. We hypothesized that the pro-adhesive properties of heparanase are mediated by its interaction with cell surface HS proteoglycans, and utilized the KKDC peptide to examine this possibility. We provide evidence that the KKDC peptide interacts with cell membrane HS, resulting in clustering of syndecan-1 and syndecan-4. We applied classical analysis of cell morphology, fluorescent and time-lapse microscopy and demonstrated that the KKDC peptide efficiently stimulates the adhesion and spreading of various cell types, mediated by PKC, Src, and the small GTPase Rac1. These results support, and further substantiate the notion that heparanase function is not limited to its enzymatic activity.

## Introduction

Proteoglycans are proteins that bear long, un-branched sugar polymers, glycosaminoglycans, which are attached to specific serine residues of the protein core [1]. Glycosaminoglycans are polymers of disaccharide units. In the case of heparan sulfate, uronic acid (either glucuronic acid or iduronic acid) and N-acetyl glucosamine repeats compose the basic structure of the proteoglycan. Despite the seemingly simple, single repeating structural motif, these sugar polymers show a great deal of structural diversity generated by complex pattern of deacetylation, sulfation, and epimerization [1]. Thus, a single heparan sulfate proteoglycan (HSPG) side chain contains distinct structural domains, composed of regions of highly sulfated, negatively-charged residues alongside of regions with modest levels of sugar modifications. Proteoglycans are abundant components of the basement membrane and extracellular matrix (ECM) of epithelium, endothelium, and connective tissues including cartilage, tendons, and bones [2], [3]. In addition, proteoglycans are also abundantly present on the cell surface, providing an important constituent of the cell's sugar coat involved in various aspects of cellular and molecular activities at the cell-ECM interface [4]. By interacting with other macromolecules such as laminin, fibronectin, and collagens I and IV, HSPGs contribute to the structural integrity, self-assembly and insolubility of the ECM and basement membrane, thus intimately modulating cell-ECM interactions [5]–[7]. ECM constituents are, however, only one class of HS-binding molecules. In fact, numerous enzymes, growth factors, cytokines and chemokines are sequestered by HSPGs on the cell surface and ECM [1], [4], [8], [9]. In general, HSPGs facilitate the biological activity of bound ligands by actively participating in receptor-ligand complex formation [10]. In other cases, HSPGs mediate cellular uptake and catabolism of selected ligands [10], or sequester polypeptides to the ECM and cell surface as an inactive reservoir [11]–[15]. Cleavage of HSPGs would ultimately release these proteins and convert them into bioactive mediators, ensuring rapid tissue response to local or systemic cues. The protein core of HSPGs is susceptible to cleavage by several classes of proteases [16], [17]. An additional mode of HSPGs cleavage is offered by the enzyme heparanase.

Heparanase is an endo-β-glucuronidase that cleaves HS side chains presumably at sites of low sulfation [18], releasing saccharide products with appreciable size (5–7 kDa) that can still associate with protein ligands and modulate their biological potency. Heparanase activity has been traditionally correlated with cell invasion associated with cancer metastasis, a consequence of structural modification that loosens the ECM barrier [19]–[21]. More recently, heparanase up-regulation was documented in an increasing number of human carcinomas and hematological malignancies [18], [22]–[25]. In many cases, heparanase induction correlated with increased tumor metastasis, vascular density, and shorter post operative survival rate, thus providing a strong clinical support for the pro-metastatic and pro-angiogenic function of the enzyme [22], [25]. In addition to the well studied catalytic feature of the enzyme, heparanase was noted to exert biological functions apparently independent of its enzymatic activity. Non enzymatic functions of heparanase include enhanced adhesion of glioma [26], lymphoma [27] and T cells [28], mediated by β1-integrin and correlated with Akt, Pyk2 and ERK activation [26], [28].

Attempting to identify functional domains that would serve as a target for drug development, we have recently identified heparin binding domains of heparanase [29]. A corresponding peptide (residues Lys^158^-Asp^171^, termed KKDC) was demonstrated to physically associate with heparin and HS, and to inhibit heparanase enzymatic activity [29]. We hypothesized that the pro-adhesive properties of heparanase are mediated by its interaction with cell surface HSPGs and utilized the unique feature of the KKDC peptide to examine this possibility.

Syndecans are a family of four transmembrane proteins capable of carrying chondroitin sulfate (CS) and HS chains. Syndecans are expressed on virtually all cell types throughout development and adulthood, and their expression can be altered under certain pathophysiological conditions, including tumor onset, progression, and metastasis [30], [31]. The presence of HS chains allows interactions with a large number of proteins, including heparin-binding growth factors, plasma proteins such as antithrombin, and extracellular matrix proteins including fibronectin. In addition, syndecans are thought to function as co-receptors, facilitating growth factors, morphogens, and integrins activity [30]–[32]. Furthermore, syndecans can associate with a large number of adaptor and signaling molecules through their cytolplasmic tail, despite being relatively small. These include ezrin, tubulin, cortactin, PDZ domain-containing proteins (i.e., syntenin), Src and PKCα [33], [34]. Syndecans emerge as central players in the interactions between the ECM and the cell surface that ultimately regulate cell spreading, adhesion, and migration. This function was largely attributed to a direct interaction of syndecans with matrix proteins such as fibronectin. In addition, clustering of syndecan-4 has been shown to initiate signaling cascades that results in PKCα and Rac1 activation that appears instrumental for cell adhesion and directional migration [33]–[37].

Here, we provide evidence that the KKDC peptide interacts with plasma membrane HS, and facilitates clustering of syndecan-1 and syndecan-4. Interestingly, while the heparanase-syndecan complex is subjected to rapid internalization and appears in endocytic vesicles short after the addition of heparanase, the KKDC peptide elicits syndecan clusters that remain on the cell surface for a relatively long period of time and fail to get internalized. We demonstrate that the KKDC peptide efficiently stimulates the adhesion and spreading of multiple cell types by activating the PKC pathway and the small GTPase Rac1. These results support and further expand the notion that heparanase function is not limited to its enzymatic activity, and highlight heparin binding domains as targets for the development of anti-cancer inhibitors.

## Materials and Methods

### Antibodies and reagents

Anti syndecan-1 monoclonal antibody (B-B4) was purchased from Serotec (Oxford, UK). Antibodies to syndecan-4 and paxillin were purchased from Santa Cruz Biotechnology (Sanata Cruz, CA). Anti-Rac 1 monoclonal antibody was purchased from Becton Dickinson Biosciences (San Diego, CA), and PAK PBD-agarose beads were from Cell Biolabs Inc (San Diego, CA). Anti-vinculin monoclonal antibody, phalloidin-TRITC, heparin, fibronectin, and the 110 kDa fibronectin-like genetically engineered protein, were purchased from Sigma (St. Louis, MO). Avidin-FITC was purchased from Vector (Burlingame, CA). The selective p38 (SB-203580), Src (PP2), Erk (PD-98059), PKC [Bisindolylmaleimide I (Bis)], Rock (Y27632) and Rac (NSC 23766) inhibitors were purchased from Calbiochem (San Diego, CA) and were dissolved in DMSO as stock solutions. DMSO was added to the cell culture as a control. The KKDC and its control, scrambled peptide were synthesized as described previously [29]. The introduced C-terminal cysteine residue mediates spontaneous peptide dimerization that can be further facilitated by enhanced oxygenation generated by stirring. For biotin labeling, an extra lysine residue was introduced at position 12 to create the sequence KKFKNSTYSRS**K** ^biotin^SVDC. This approach was undertaken in order to preserve the N-terminal lysine residues critical for the peptide interaction with HS [29]. A peptide from the COOH-terminal heparin-binding domain of fibronectin (Hep II peptide, WQPPRARI) [38] was purchased from Sigma (St. Louis, MI).

### Cells and cell culture

U87 MG human glioma and Colo 320 colon carcinoma cells were purchased from the American Type Culture Collection (ATCC). Rat C6 glioma cells were kindly provided by Dr. Eli Keshet (The Hebrew University Hadassah Medical School, Jerusalem) [39], and human skin fibroblasts were kindly provided by Dr. H. Mendel (Rambam Health Care Campus, Haifa). ARH-77 leukemia-derived F cells were kindly provided by Dr. Ben-Zion Katz (Soraski Medical Center, Tel-Aviv, Israel). F cells are a sub-population of the ARH-77 cell line which poorly adhere and mainly floats (F) once plated on fibronectin-coated dishes [40]. Cells were grown in Dulbecco's Modified Eagle's Medium (DMEM) supplemented with glutamine, pyruvate, antibiotics and 10% fetal calf serum in a humidified atmosphere containing 5% CO_2_ at 37°C. Wild type Chinese hamster ovary (CHO) K1 and mutant cells (pgs A-745) deficient in xylosyltransferase and unable to initiate glycosaminoglycan synthesis, were kindly provided by Dr. Jeffery Esko (University of California, San Diego), and grown in RPMI 1640 medium supplemented with 10% FCS and antibiotics [39]. Recombinant wild type and enzymatically-inactive heparanase mutated at glutamic acid residues 225 and 343 [double mutant (DM)] critical for enzymatic catalysis [41] were purified from the conditioned medium of HEK 293 transfected cells, essentially as described [42].

### Cell morphology and immunocytochemistry

Cells were plated on glass cover slips uncoated or coated with fibronectin or the 110 kDa fibronectin-like protein for the time indicated in the absence (Con) or presence of the indicated peptides [scrambled (Scr), or KKDC; (50 µg/ml)], proteins [latent 65 kDa or mutated, enzymatically inactive heparanase (1 µg/ml)], or inhibitors. Cells were then fixed with 4% paraformaldehyde in PBS for 15 minutes, and visualized. For immunofluorescent staining, cells were fixed with cold methanol for 10 minutes, washed with PBS and subsequently incubated in PBS containing 10% normal goat serum for 1 hour at room temperature, followed by 2 hours incubation with the indicated primary antibody. Cells were then extensively washed with PBS and incubated with the relevant Cy2/Cy3-conjugated secondary antibody (Jackson ImmunoResearch, West Grove, PA) for 1 hour, washed and mounted (Vectashield, Vector, Burlingame, CA). Staining was observed under a fluorescent confocal microscope. For actin staining, cells were fixed with 4% paraformaldehyde, permeabilized with 0.5% triton X-100 for 2 minutes, washed and incubated with TRITC-phalloidin (Sigma) for 30 minutes and visualized by confocal microscopy, as described [43], [44].

### Cell lysates and protein blotting

Cell cultures were incubated for 24 hours under serum-free conditions, pretreated with 1 mM orthovanadate for 10 minutes at 37°C, washed twice with ice cold PBS containing 1 mM orthovanadate and scraped into lysis buffer (50 mM Tris-HCl, pH 7.4, 150 mM NaCl, 1% Triton-X100, 1 mM orththovanadate, 1 mM PMSF) containing a cocktail of protease inhibitors (Roche, Mannheim, Germany). Total cellular protein concentration was determined by the BCA assay, according to the manufacturer's (Pierce, Rockford, IL) instructions. Thirty micrograms of cellular protein were subjected to SDS polyacrylamid gel (SDS-PAGE) and immunoblotting, as described [29], [45]. For estimation of Rac1 activation, lysis buffer included 50 mM Tris-HCl, pH 7.4, 300 mM NaCl, 10 mM MgCl, 4% glycerol, and 1% Triton X-100. The amount of GTP-bound Rac1 was analyzed by incubating total cell lysates (200 µg) with 20 µg of the p21-binding domain (PBD) of PAK-agarose beads. Following 30 minutes incubation, the beads were washed and, after electrophoresis and blotting, membranes were probed with anti-Rac1 antibodies [26].

### Time Lapse microscopy

Cells were grown on Corning/Nunc 6 wells dishes in DMEM medium and viewed with inverted motorized microscope (DMIRE2 Leica, Germany). Images were captured using cooled B/W CCD camera (Retiga EXI, Qimaging, USA). Time-lapse acquisition was made with custom written software (Provided by Uri Alon, Weizmann Institute, Rehovot, Israel), inserted into Image-Pro software (MediaCybernetics, USA). The system is equipped with an on stage incubator (Cube & Box, LIS, Switzerland).

## Results

### The KKDC peptide interacts with the plasma membrane

**Figure 1:**
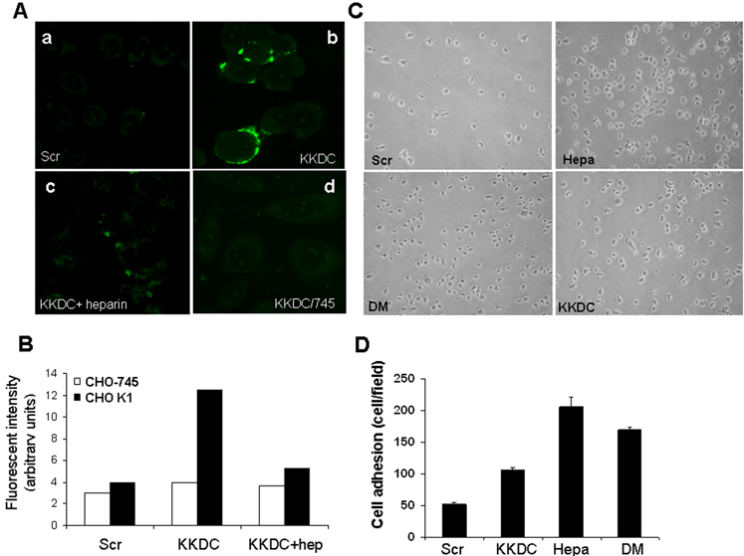
The KKDC peptide interacts with plasma membrane heparan sulfate. A,B: peptide binding. Wild type (CHO K1; a–c) and heparan sulfate-deficient (CHO-745; d) cells were incubated (2 hours) with biotinylated KKDC (b–d) or control peptide (Scr; a) in the absence (a, b, d) or presence (c) of 10 µg/ml heparin. Cells were then washed, fixated with 4 % paraformaldehyde and bound peptide was visualized with avidin-FITC and fluorescence microscopy (A) or analyzed by FACS Scan (B). C, D Heparanase and the KKDC peptide promotes cell adhesion. Leukemia-derived ARH-77 cells that were selected to grow in suspension (F cells), were plated on fibronectin coated plates for 30 minutes in the presence of heparanase (Hepa; 1 µg/ml), inactive heparanase (DM; 1 µg/ml), KKDC peptide (50 µM), or control peptide (Scr, 50 µM). Plates were gently washed and cell adhesion was visualized by light microscopy (C). Adherent cells were counted and quantified (D).

We have identified heparin binding domains that mediate the interaction of heparanase with its HS substrate and demonstrated that a peptide corresponding to Lys^158^-Asn^171^ (KKDC), inhibits heparanase enzymatic activity [29]. Although the KKDC peptide has been shown to physically interact with heparin and HS [29], binding and localization at the cellular level have not been so far demonstrated. For this purpose, the KKDC and control, scrambled peptides were labeled with biotin on an extra lysine residue introduced in order to maintain the N-terminal lysine residues critical for HS binding [29] intact, and peptide binding was detected by Avidin-FITC. No detectable binding of the control, scrambled peptide to CHO K1 cells was observed (Fig. 1A, Scr). In contrast, the KKDC peptide gave rise to a punctuated, readily detected staining (Fig. 1A, KKDC) that was significantly reduced by the addition of heparin (Fig. 1A, KKDC+heparin). Furthermore, no staining was detected upon addition of the biotinylated KKDC peptide to CHO-745 cells deficient of HSPGs (Fig. 1A, KKDC/745), suggesting that the KKDC peptide interacts with cell surface HSPGs. Interaction of the KKDC peptide with cell membrane molecules, lack of interaction with CHO-745 cells, and heparin competition were further demonstrated by FACS analysis (Fig. 1B). Thus, the KKDC peptide enables to elucidate the effect of heparanase-HS interaction on cell behavior, excluding other classes of heparanase binding proteins such as low density lipoprotein receptor-related protein (LRP) and the mannose 6-phosphate receptor (MPR) [46].

### Inactive heparanase facilitates leukemia cell adhesion

We have previously noted that over expression of heparanase enhances the adhesion of mouse Eb lymphoma cells to ECM and endothelial cells. Augmented cell adhesion was maintained by cells transfected with point mutated, inactive heparanase, or in the presence of laminaran sulfate, an inhibitor of heparanase enzymatic activity [27], arguing for enzymatic-independent function. Similarly, exogenous addition of purified latent 65 kDa heparanase to poorly adhesive human leukemia ARH-77 F cells, selected to grow in suspension [40], significantly enhanced their adhesion to fibronectin (Fig. 1C&D, Hepa). Moreover, addition of recombinant, enzymatically inactive double mutated (DM) heparanase facilitated F cells adhesion similar in magnitude to the wild type protein (Fig. 1C&D, DM), further supporting the notion that improved cellular adhesion upon heparanase over-expression [27] or exogenous addition (Fig. 1) does not involve enzymatic aspects.

The scope of heparanase function has recently been challenged by evidence documenting its ability to activate signaling molecules (i.e., Akt, p38, Src) in an enzymatic-independent manner, resulting in enhanced gene transcription [42], [47] and cell adhesion [26]–[28]. Enhanced cell adhesion by heparanase may be due to binding and activation of plasma membrane proteins such as syndecans, a class of trans-membrane HSPGs shown to modulate adhesion complexes [30], [31], [34]. In order to examine this possibility, we took advantage of the specific feature of the KKDC peptide and exposed ARF-77 F cells to the KKDC peptide or its control, scrambled counterpart. Indeed, the KKDC peptide elicited a significant, two-fold increase in F cells adhesion (Fig. 1C&D, KKDC), while the control, scrambled peptide had no such effect (Fig. 1C, Scr). These results suggest that interaction between heparanase and plasma membrane HSPGs is sufficient to enhance cell adhesion.

### The KKDC peptide induces syndecan clustering and enhances cell spreading

**Figure 2:**
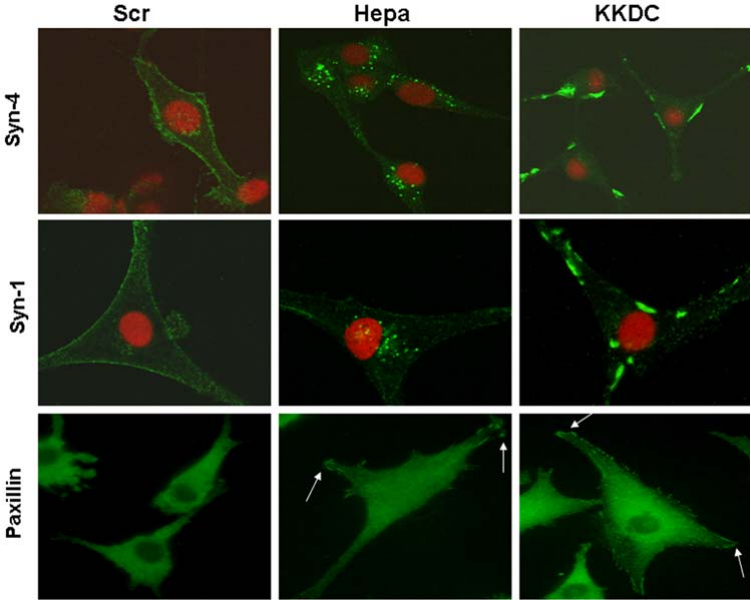
The KKDC peptide induces syndecan clustering and enhances cell spreading. U87 cells were serum starved for 24 hours and were then incubated with the KKDC/Scr peptides (50 µM) or heparanase (1 µg/ml) for 1 hour. Cells were then gently washed, fixed with cold methanol and subjected to fluorescent staining with anti-syndecan-4 (upper panels) or anti-syndecan-1 (middle panels) antibodies. U87 cells were also plated on the 110 kDa fibronectin-like protein and incubated for 4 hours in the presence of KKDC/Scr peptides (50 µM) or heparanase (1 µg/ml). Cells were then washed, fixed with 4% paraformaldehyde and stained with anti-paxillin antibodies (lower panels).

Syndecan-4 appears regularly spread in the plasma membrane of U87 glioma cells (Fig. 2, upper panels, Scr). In contrast, syndecan-4 was mostly localized to perinuclear endocytic vesicles following heparanase addition (Fig. 2, upper panels, Hepa), co-localizing with heparanase [39]. Interestingly, however, syndecan-4 was not internalized upon the addition of the KKDC peptide but rather appeared clustered on the cell surface (Fig. 2, upper panels, KKDC). A similar localization was noted for syndecan-1 (Fig. 2, middle panel, KKDC). Next, we examined U87 cell spreading upon stimulation with heparanase or the KKDC peptide by staining the cells for paxillin, a well-characterized focal-adhesion component. Cells treated with heparanase (1 µg/ml) appeared better spread and focal adhesion complexes were nicely decorated by paxillin (Fig. 2, lower panels, Hepa, arrows). Enhanced cell spreading was even more pronounced in the presence of the KKDC peptide (50 µM), where multiple focal contacts appeared (Fig. 2, lower panels, KKDC), indicating that syndecans clustering by the KKDC peptide facilitates cellular spreading. These observations were further confirmed by employing several additional cell adhesion systems.

**Figure 3:**
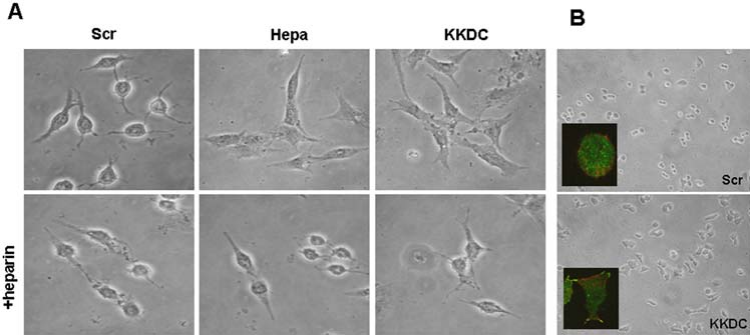
The KKDC peptide induces cell spreading. A. C6 glioma cells were serum-starved for 24 hours, and were then incubated with heparanase (1 µg/ml), KKDC or control (Scr) peptides (50 µM) for 18 hours in the absence (upper panels) or presence of heparin (50 µg/ml; lower panels). Cell spreading was evaluated by light microscopy. B. Colo320 colon carcinoma cells were plated on fibronectin for 1 hour followed by incubation with the KKDC (lower panel) or the control (Scr; upper panel) peptides (50 µM) for additional 18 hours. Cell spreading was evaluated by light microscopy and by staining for paxillin (insets).

Rat C6 glioma cells rapidly adhere and spread once plated on tissue culture plastic dish. The cells, however, assume a round morphology once cultured in serum-free medium, and remained round following the addition of control peptide (Fig. 3A, Scr; Movie S1). In striking contrast, cells appeared nicely spread following the addition of heparanase (Fig. 3A, Hepa; Movie S2) and even more so the KKDC peptide (Fig. 3A, KKDC; Movie S3), an effect that was completely inhibited by heparin (Fig. 3A, lower panels; Movie S4). Similarly, treatment of Colo 320 colon carcinoma cells with the KKDC peptide enhanced cell spreading (Fig. 3B, KKDC) and focal complexes formation evident by paxillin staining (Fig. 3B, KKDC, inset).

**Figure 4:**
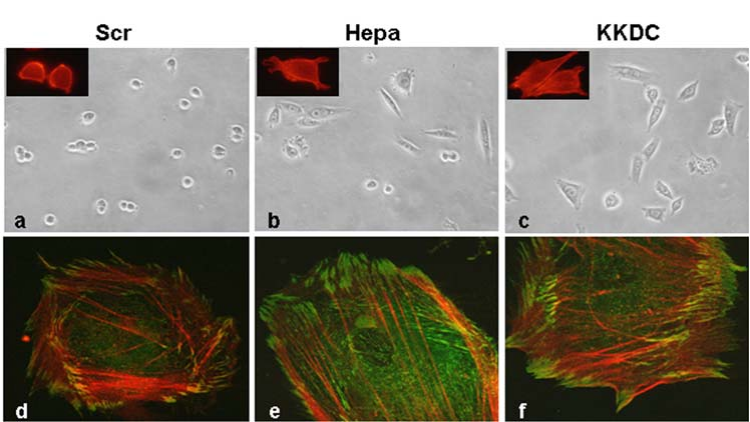
Heparanase and the KKDC peptide enhance cell spreading and formation of actin stress fibers. CHO cells (a–c) and human fibroblasts (d–f) were plated on the 110 kDa fibronectin-like protein and incubated with heparanase (Hepa; 1 µg/ml) or the KKDC/Scr peptides (50 µM) for 18 hours (a–c) or 1 hour (d–f). Cells were then visualized by light microscopy (a–c) or fixed with 4% paraformaldehyde and stained with phalloidin-TRITC (a–c, insets) or double stained with phalloidin-TRITC (red) and anti-vinculin (green) antibody (d–e). Cells treated with heparanase (b,e) or with the KKDC peptide (c,f) appeared spread and formed more focal adhesions (e,f).

Fibronectin is a multifunctional glycoprotein that mediates a number of important biological functions including cell adhesion and migration [48]. The diverse biological activities attributed to fibronectin have been localized to specific molecular domains. Thus, the main cell binding, RGD-rich domain is localized within the 10^th^ type III module, while heparin binding is mediated by modules 12–14 [49]. Fibronectin-like protein polymer has been engineered to include 13 copies of the RGD-rich sequence, resulting in a protein with a stable three-dimensional conformation and molecular mass of 110 kDa. CHO K1 cells plated on dishes coated with the 110 kDa fibronectin-like protein adhered, but failed to spread and appeared round in morphology (Fig. 4, Scr, upper panel), as expected [32]. In contrast, CHO K1 cells plated on the fibronectin-like protein in the presence of heparanase (Fig. 4, Hepa) or the KKDC peptide appeared elongated and more spread (Fig. 4, upper panels). In addition, cells treated with heparanase exhibited typical membrane protrusions (Fig. 4, inset), and actin stress fibers were evident in cells treated with the KKDC peptide (Fig. 4, inset). Similarly, enhanced stress fiber and focal complexes formation, evident by phalloidin and vinculin staining, respectively, were noted in human fibroblasts plated on the 110 kDa fibronectin-like polymer substrate and treated with heparanase or the KKDC peptide (Fig. 4, lower panels). Collectively, these results suggest that syndecan clustering by heparanase or the KKDC peptide facilitates cell adhesion and spreading and can substitute, to some extent, the heparin binding domains of fibronectin.

### Enhanced cell spreading by heparanase/KKDC is mediated by PKC, Src, and Rac1

**Figure 5:**
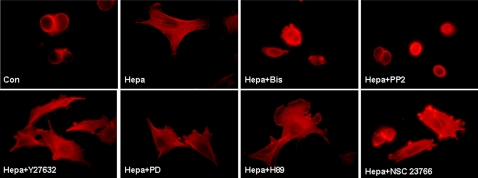
Cell spreading induced by heparanase is mediated by PKC, Rac, and Src. CHO cells were plated on the 110 kDa fibronectin-like protein, without (Con) or with heparanase, in the absence (Hepa) or presence of selective PKC (Bis), Src (PP2), ROCK (Y27632), MAPK (PD), PKA (H89), and Rac (NSC 23766) inhibitors for 6 hours. Cells were then fixed with 4% paraformaldehyde and stained with phalloidin-TRITC.

**Figure 6:**
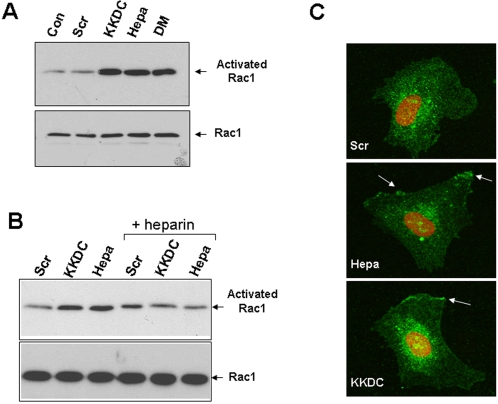
Heparanase and the KKDC peptide induce Rac1 activation. A. F cells were washed with serum free medium and were then treated for 30 minutes with the KKDC/Scr peptides (50 µM), wild type heparanase (Hepa) or inactive double mutant heparanase (DM) in serum free medium. Cell lysates (100 µg) were then subjected to pull-down with GST-PAK-agarose beads for detection of active Rac1 (upper panels), as described in “Materials and Methods”. Cell lysates (30 µg) were subjected to immunoblotting with anti-Rac1 antibody (lower panels). B. U87 cells were kept in serum-free medium for 24 hours and were then stimulated with the KKDC/Scr peptides (50 µM) or heparanase (Hepa; 1 µg/ml) for 30 min without or with heparin (50 µg/ml; +heparin). Rac activation was then evaluated as above. C. Human umbilical vein endothelial cells (HUVEC) were plated on the 110 kDa fibronectin-like protein and were incubated with the KKDC/Scr peptides (50 µM) or heparanase (Hepa; 1 µg/ml) for 3 hours. Cells were then fixed and stained with anti-Rac1 antibody. Note Rac1 localization to the plasma membrane (arrows) following treatment with heparanase or the KKDC peptide.

As shown above, CHO K1 cells depend on heparanase or the KKDC peptide for spreading on the 110 kDa fibronectin-like substrate (Fig. 4). We, next, utilized this cell system to examine signaling pathways that mediate the effect of heparanase. To this end, cells were plated on the 110 kDa fibronectin-like substrate without (Con, Fig. 5) or with heparanase (Fig. 5, Hepa), or pretreated with the indicated inhibitor prior to heparanase addition, and cell spreading was then examined. As previously noted, CHO K1 cells spreading was markedly enhanced in the presence of heparanase (Fig. 5, Hepa). Cell spreading stimulated by heparanase was not significantly altered by inhibitors of MAPK (Fig. 5, Hepa+PD), PKA (Fig. 5, Hepa+H89), or ROCK, a down stream effector of RhoA (Fig. 5, Hepa+Y27632). In contrast, cell spreading stimulated by heparanase was significantly attenuated by Src (Fig. 5, Hepa+PP2), PKC (Fig 5, Hepa+Bis), and Rac1 (Fig. 5, Hepa+NSC 23766) inhibitors. The small GTPase Rac1 is thought to play instrumental roles in the early phases of cell adhesion and spreading [50]. Thus, initial membrane protrusion is governed by Cdc42 and Rac1 activation, resulting in filopodia/lamellipodia extensions and focal complexes formation. Subsequent activation of RhoA induces the maturation of focal complexes to focal adhesions, assembly of stress fibers, and cell locomotion [50]. Given the observed enhancement of cell adhesion and spreading upon heparanase/KKDC treatment, we examined RhoA and Rac1 activation by employing agarose beads coupled to RhoA-binding domain of Rhotekin, and the p-21 binding domain (PBD) of p21-activated protein kinase (PAK) that specifically binds the active, GTP-bound form of RhoA and Rac1, respectively. Rac1 activation was markedly induced following heparanase addition to ARF-77 leukemia F cells (Fig. 6A, Hepa, upper panel). Double-mutated, inactive heparanase (DM) and the KKDC peptide stimulated Rac1 activation to the same extent (Fig. 6A, DM, upper panel) or even higher (KKDC) than wild type heparanase (Fig. 6A, upper panel). Similarly, a significant activation of Rac1 was noted in U87 glioma cells stimulated with heparanase or the KKDC peptide (Fig. 6B, upper panel), activation that was abolished by the addition of heparin (Fig. 6B+heparin), while RhoA activation was not evident (not shown). Moreover, Rac1 was noted to assume membrane localization following heparanase or KKDC stimulation (Fig. 6C), a cellular translocation that is considered essential for the activation of down stream effectors, and cell migration [51].

Altogether, the results indicate that syndecan activation by soluble heparanase is sufficient to stimulate cell adhesion, function that clearly does not require heparanase enzymatic activity and is efficiently mimicked by the KKDC peptide.

## Discussion

**Figure 7:**
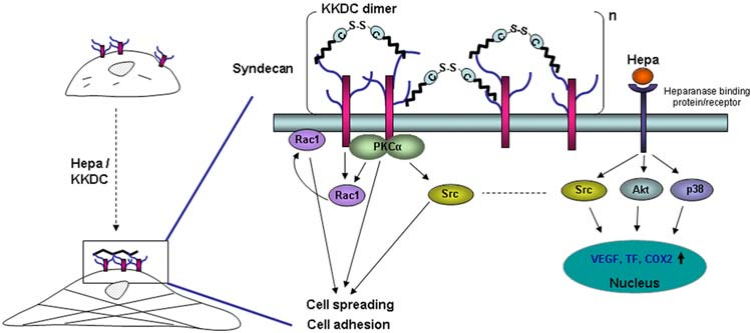
A schematic diagram of HSPG activation by heparanase/KKDC. Clustering of syndecan family members, and possibly glypicans, by the KKDC dimer or the two heparin binding domains of heparanase facilitates cell-adhesion and cell-spreading (left). Enhanced cell adhesion and spreading is mediated by the recruitment and activation of PKCα, Rac1, and Src. In addition, heparanase is thought to interact with heparanase-binding cell surface protein/receptor, leading to HS-independent Akt activation. Similarly, p38 and Src activation by heparanase may also be mediated by this receptor, resulting in enhanced transcription of genes such as vascular endothelial growth factor (VEGF) [42], tissue factor (TF) [47], or Cox2 [60], and further contribute to cell adhesion, spreading and motility (right).

We have previously noted that heparanase facilitates the adhesion, spreading, and migration of several cell types in a manner that appeared independent of its enzymatic activity [26]–[28], [52]. We have also noted that heparanase is internalized as a complex with syndecan family members [39], and induces Rac1 activation [26], yet the causal link between these observations was unclear. The results presented in this study merge these findings in a linear mode in which syndecan clustering by heparin binding domains of heparanase initiates signaling cascades that involve Rac1, Src, and the PKC pathways, resulting in enhanced cell adhesion and spreading (Fig. 7). Syndecan clustering and activation are likely mediated by the two functional heparin binding domains of heparanase (Lys^158^-Asp^171^ and Gln ^270^-Lys^280^) [29], which are mimicked by the KKDC peptide dimer. These results not only provide a molecular basis for non-enzymatic function of heparanase, but also suggest a novel mechanism by which syndecan molecules are activated. Thus, while syndecan clustering is thought to be mediated physiologically by ECM resident fibril fibronectin, or artificially engaged by antibodies [37], our results suggest that syndecans can also be activated by soluble ligands such as heparanase.

A key tool for deciphering this mode of heparanase function is provided by the KKDC peptide. A cysteine residue introduced at the peptide C-terminus mediates spontaneous peptide dimerization that can be further facilitated by enhanced oxygenation, a procedure shown to improve the interaction of peptides with heparin [53], yielding a two-headed molecule with intact heparin binding domains at both ends (Fig. 7). Indeed, the addition of ethandithiol (EDT) which binds covalently to the sulfate group of cysteine and prevents di-sulfide bridge formation between cysteine residues, markedly inhibited the interaction of the KKDC peptide with heparin (Fig. S1A) and its anti-heparanase activity (not shown). Thus, the peptide dimer and its two-headed heparin binding domains mimic the two functional heparin binding domains of heparanase [29], and enables syndecan clustering (Fig. 7). In fact, syndecan clusters elicited by the KKDC peptide appeared exceptionally large and failed to get internalized at the time points examined, while the heparanase-syndecan complex is rapidly and efficiently internalized (Fig. 2) [39]. The ability of the KKDC peptide to interact with heparin [29] and to cluster syndecans (Fig. 2) appears unique, since peptides derived from two additional domains in heparanase containing a Cardin-Weintraub consensus sequences and suspected to mediate its interaction with heparan sulfate (Gln^270^-Lys^280^ and Lys^411^-Arg^432^) exhibited only a very weak interaction with heparin and failed to inhibit heparanase enzymatic activity [29]. Thus, the mere presence of Cardin-Weintraub consensus sequences [9] appears not sufficient to ensure bioactive peptide, further highlighting the unique feature of the KKDC peptide. Moreover, a peptide derived from the C-terminal heparin-binding domain of fibronectin failed to facilitate the adhesion of ARH-77 leukemia-derived F cells, compared with a significant pro-adhesive effect exerted by the KKDC peptide (Fig. S2 and data not shown). Notably, cells treated with the KKDC peptide appeared better spread and formed more focal contacts compared with cells treated with heparanase. This was evident by the morphology of C6 and Colo 320 cells (Fig. 3), paxillin staining of U87 glioma (Fig. 2) and Colo 320 cells (Fig. 3B), and phalloidin and vinculin staining of CHO and human skin fibroblasts plated on the 110 kDa fibronectin-like substrate (Fig. 4), indicating that syndecan clustering by the KKDC peptide is translated biochemically to cellular response. In contrast, heparanase appeared more efficient in stimulating the adhesion of floating ARH-77 leukemia cells compared with the KKDC peptide (Fig. 1). This result indicates that syndecan activation alone by the KKDC peptide may not be sufficient for efficient cell adhesion, and that cooperation with additional adhesion molecules such as integrins is required [32]. Integrin activation was noted upon heparanase over expression or exogenous addition [26], [28]. This activation does not seem to involve enzymatic aspects since mutated, inactive heparanase (DM) was as potent as wild type heparanase in facilitating ARH-77 F cell adhesion (Fig. 1). The molecular mechanism underlying integrin activation by heparanase awaits detailed characterization.

The prime syndecan candidate for mediating the effect of heparanase/KKDC on cell adhesion is syndecan-4 since this molecule is ubiquitously expressed and is the only syndecan that appears to reside at, and exhibit a functional role in the assembly of focal adhesions [31], [34], [54]. The KKDC peptide, however, clustered efficiently both syndecan-1 and syndecan-4 (Fig. 2) and possibly additional syndecan family members, raising the possibility that enhanced cell adhesion and spreading is not solely mediated by syndecan-4. Indeed, essentially all syndecan family members are implicated in cell adhesion. For example, over expression of syndecan-1 in syndecan deficient Raji lymphoblastoid or COS-7 cells stimulated cell spreading [30]. Similarly, syndecan-2 was noted to promote focal adhesion formation in Lewis-lung carcinoma cells [31], and syndecan-2 and syndecan-3 induce filopodia formation in COS-1, CHO-K1, Swiss 3T3, and dendritic cells [30], [33], [55]. It is therefore likely that heparanase/KKDC induce the clustering of more than one syndecan family member and, possibly, also of glypicans, leading to enhanced cell adhesion and spreading. This possibility is further supported by the localization of syndecans following the addition of heparanase or the KKDC peptide. Clusters of syndecan-1 and syndecan-4 were similarly distributed on the cell surface following addition of the KKDC peptide (Fig. 2) in a pattern that does not coincide with focal complexes. Rapid internalization of heparanase-syndecan complexes following heparanase addition clearly implies that enhanced cell adhesion does not require localization of syndecans at focal adhesions, but rather involves syndecan activation.

Syndecan activation by soluble ligands such as heparanase may also have important clinical implications. Over expression of syndecan-1 has been observed in pancreatic, gastric and breast carcinomas, correlating with increased tumor aggressiveness and poor clinical prognosis, while syndecan-2 is often over expressed in colon carcinoma, and syndecan-4 is up-regulated in hepatocellular carcinoma [30], coinciding with heparanase up-regulation in these carcinomas [22], [25]. Syndecan-1 is particularly abundant in multiple myeloma [56], where an emerging role for heparanase has recently been shown [57]–[59]. Thus, elevated levels of heparanase may activate syndecan family members and promote tumor metastasis and angiogenesis, resulting in poor prognosis often associated with patients exhibiting high levels of heparanase [22], [25].

Taken together, the results clearly highlight heparanase as a multi functional protein, exhibiting enzymatic activity-dependent and -independent functions. Better understanding of the basic biology of this protein will contribute to the development of efficient heparanase inhibitors directed, for example, against the heparin binding domains of heparanase [29], [44]. Monoclonal antibodies or small molecules directed against this domain will not only inhibit heparanase enzymatic activity [44], but will also suppress syndecans activation, resulting in a more efficient neutralization of heparanase functions. On the other hand, the anti-cancer efficacy of the KKDC peptide which inhibits heparanase enzymatic activity [44] (Fig. S1B) is questionable taking into account its pro-adhesive properties.

## Supporting Information

Movie S1 Time lapse microscopy. Rat C6 glioma cells (2×104) were plated in a 6-well plate in complete growth medium, followed by 20 hours incubation in serum-free medium. Cells were then incubated with control scrambled peptide. Six fields in each well were randomly selected, and were examined every 10 min for 10 hours by the time lapse system. Representative time lapse movie is shown.
(6.15 MB AVI)

Movie S2 Time lapse microscopy. Rat C6 glioma cells (2×104) were plated in a 6-well plate in complete growth medium, followed by 20 hours incubation in serum-free medium. Cells were then incubated with heparanase (1 µg/ml); Six fields in each well were randomly selected, and were examined every 10 min for 10 hours by the time lapse system. Representative time lapse movie is shown.
(7.14 MB AVI)

Movie S3 Time lapse microscopy. Rat C6 glioma cells (2×104) were plated in a 6-well plate in complete growth medium, followed by 20 hours incubation in serum-free medium. Cells were then incubated with the KKDC peptide; Six fields in each well were randomly selected, and were examined every 10 min for 10 hours by the time lapse system. Representative time lapse movie is shown.
(6.99 MB AVI)

Movie S4 Time lapse microscopy. Rat C6 glioma cells (2×104) were plated in a 6-well plate in complete growth medium, followed by 20 hours incubation in serum-free medium. Cells were then incubated with the KKDC peptide with heparin (10\µg/ml); Six fields in each well were randomly selected, and were examined every 10 min for 10 hours by the time lapse system. Representative time lapse movie is shown.
(6.15 MB AVI)

Figure S1 KKDC peptide dimerization significantly improves heparin binding and anti-heparanase properties. A. Heparin binding. KKDC peptide was synthesized in the absence (KKDC) or presence of ethandithiol (EDT), which binds covalently to the sulfate group of cysteine and prevents di-sulfide bridge formation between cysteine residues. Peptides (50 µM) were incubated (2 hours, 4°C) with heparin-Sepharose beads in PBS, washed with PBS supplemented with NaCl to a final concentration of 0.35 M, followed by one wash with PBS. Dye-free sample buffer was added and the beads were boiled for 5 minuntes, centrifuged and the supernatants were loaded on Tris-Tricine gel. Subsequently, gels were stained with Coomassie blue to visualize bound peptides (arrow). B. Heparanase enzymatic activity. B16 melanoma cells (2×106) were resuspended in RPMI medium and incubated (18 hours, 37°C) with 35S-labeled ECM in the absence (filled rectangle) or presence of control scrambled peptide dimer (green rectangle; 50 µM), KKDC peptide undergoing spontaneous dimerization (filled triangle; 50 µM), KKDC peptide following enhanced dimerization (blue rectangle; 50 µM), or heparin (filled circle; 15 µg/ml). The incubation medium (1 ml) containing sulfate labeled degradation fragments was subjected to gel filtration on a Sepharose CL-6B column. Fractions (0.2 ml) were eluted with PBS and their radioactivity counted in a beta-scintillation counter. Degradation fragments of HS side chains are eluted at 0.5<Kav<0.8 (peak II, fractions 15–40) and represent heparanase degradation products.
(0.61 MB TIF)

Figure S2 Leukemia-derived ARH-77 cells that were selected to grow in suspension (F cells), were plated on gelatin coated plates for 30 minutes in the presence of the KKDC (50 µM), Hep II (100 µM), or control (Scr) peptide (50 µM). Plates were then gently washed and cell adhesion was visualized by light microscopy.
(0.31 MB TIF)
