# Supplementary figures and images for: Heparanase Facilitates Cell Adhesion and Spreading by Clustering of Cell Surface Heparan Sulfate Proteoglycans
Source: PLoS One. 2008 Jun 11;3(6):e2319. doi: 10.1371/journal.pone.0002319 (PMC2405934; doi:10.1371/journal.pone.0002319)

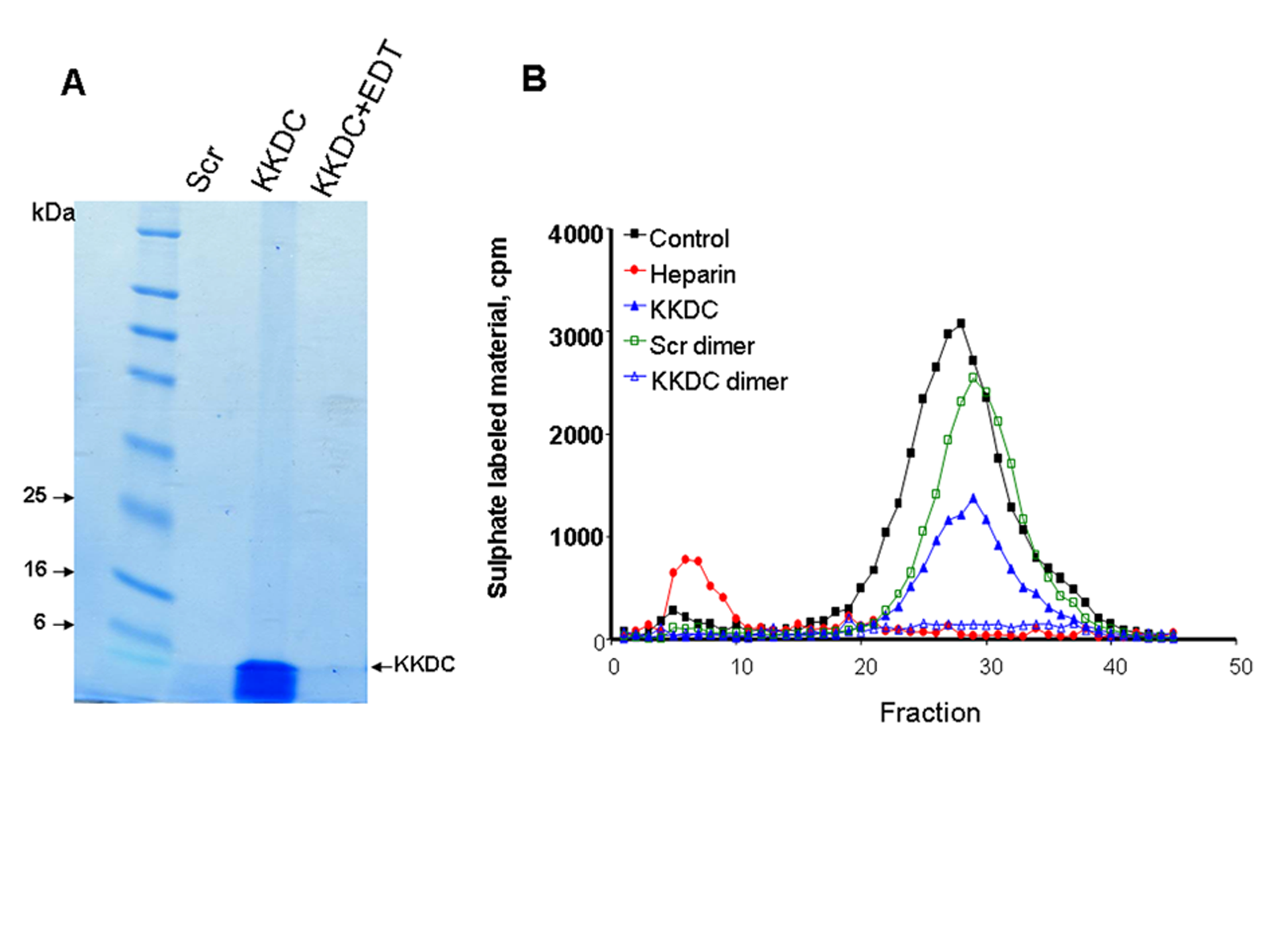

Supplement: Figure S1 — KKDC peptide dimerization significantly improves heparin binding and anti-heparanase properties. A. Heparin binding. KKDC peptide was synthesized in the absence (KKDC) or presence of ethandithiol (EDT), which binds covalently to the sulfate group of cysteine and prevents di-sulfide bridge formation between cysteine residues. Peptides (50 µM) were incubated (2 hours, 4°C) with heparin-Sepharose beads in PBS, washed with PBS supplemented with NaCl to a final concentration of 0.35 M, followed by one wash with PBS. Dye-free sample buffer was added and the beads were boiled for 5 minuntes, centrifuged and the supernatants were loaded on Tris-Tricine gel. Subsequently, gels were stained with Coomassie blue to visualize bound peptides (arrow). B. Heparanase enzymatic activity. B16 melanoma cells (2×10^6^) were resuspended in RPMI medium and incubated (18 hours, 37°C) with 35S-labeled ECM in the absence (filled rectangle) or presence of control scrambled peptide dimer (green rectangle; 50 µM), KKDC peptide undergoing spontaneous dimerization (filled triangle; 50 µM), KKDC peptide following enhanced dimerization (blue rectangle; 50 µM), or heparin (filled circle; 15 µg/ml). The incubation medium (1 ml) containing sulfate labeled degradation fragments was subjected to gel filtration on a Sepharose CL-6B column. Fractions (0.2 ml) were eluted with PBS and their radioactivity counted in a beta-scintillation counter. Degradation fragments of HS side chains are eluted at 0.5<Kav<0.8 (peak II, fractions 15–40) and represent heparanase degradation products. (0.61 MB TIF) [file pone.0002319.s005.tif]

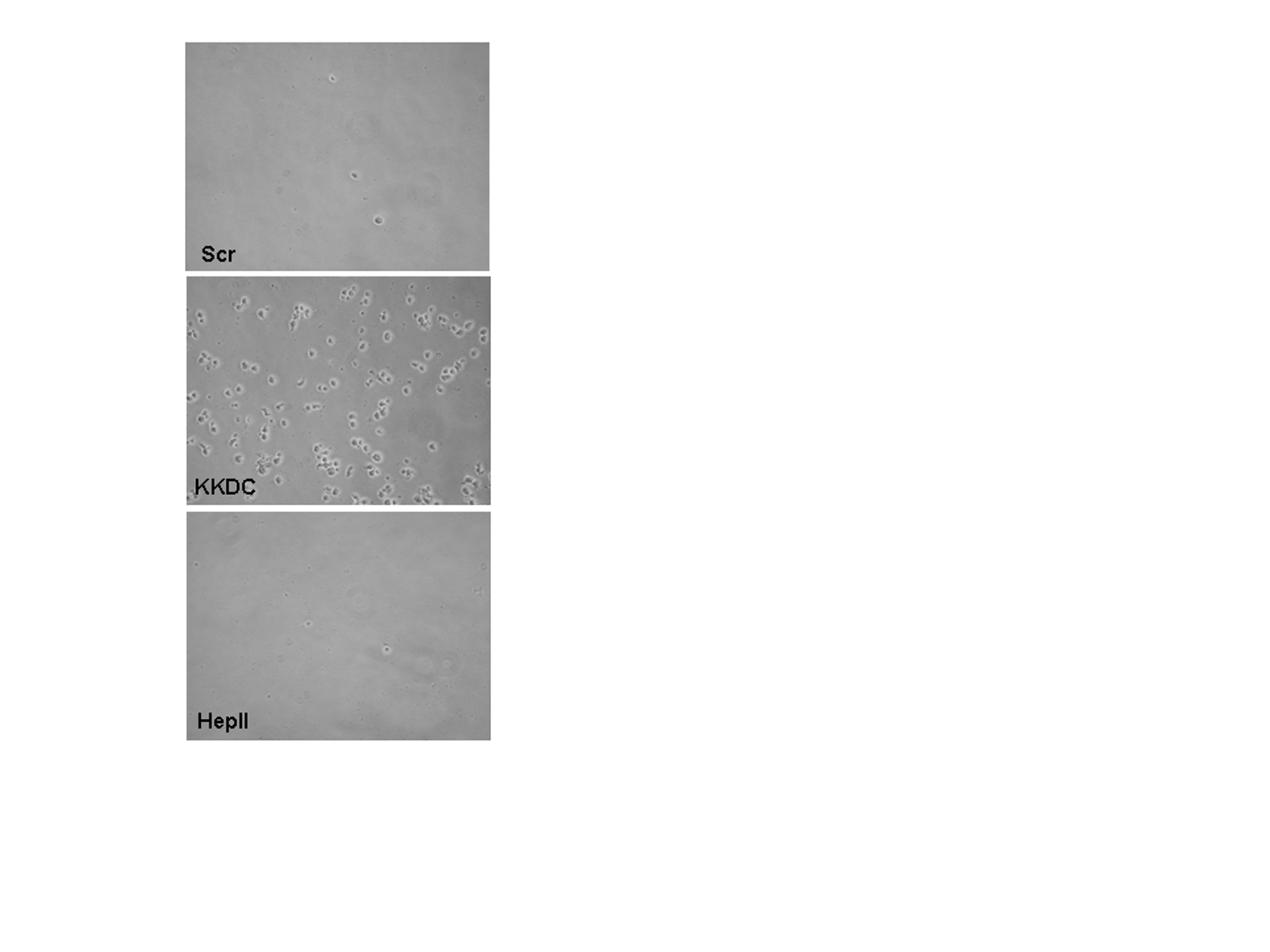

Supplement: Figure S2 — Leukemia-derived ARH-77 cells that were selected to grow in suspension (F cells), were plated on gelatin coated plates for 30 minutes in the presence of the KKDC (50 µM), Hep II (100 µM), or control (Scr) peptide (50 µM). Plates were then gently washed and cell adhesion was visualized by light microscopy. (0.31 MB TIF) [file pone.0002319.s006.tif]
